# Tissue Tregs and Maintenance of Tissue Homeostasis

**DOI:** 10.3389/fcell.2021.717903

**Published:** 2021-08-18

**Authors:** Qing Shao, Jian Gu, Jinren Zhou, Qi Wang, Xiangyu Li, Zhenhua Deng, Ling Lu

**Affiliations:** ^1^Hepatobiliary Center, The First Affiliated Hospital of Nanjing Medical University, Nanjing, China; ^2^Research Unit of Liver Transplantation and Transplant Immunology, Chinese Academy of Medical Sciences, Nanjing, China; ^3^Jiangsu Cancer Hospital, Jiangsu Institute of Cancer Research, Nanjing Medical University Affiliated Cancer Hospital, Nanjing, China; ^4^Jiangsu Key Laboratory of Cancer Biomarkers, Prevention and Treatment, Collaborative Innovation Center for Cancer Personalized Medicine, Nanjing Medical University, Nanjing, China

**Keywords:** regulatory T cells, tissue Tregs, transcription, immune homeostasis, immune maintenance

## Abstract

Regulatory T cells (Tregs) specifically expressing Forkhead box P3 (Foxp3) play roles in suppressing the immune response and maintaining immune homeostasis. After maturation in the thymus, Tregs leave the thymus and migrate to lymphoid tissues or non-lymphoid tissues. Increasing evidence indicates that Tregs with unique characteristics also have significant effects on non-lymphoid peripheral tissues. Tissue-resident Tregs, also called tissue Tregs, do not recirculate in the blood or lymphatics and attain a unique phenotype distinct from common Tregs in circulation. This review first summarizes the phenotype, function, and cytokine expression of these Tregs in visceral adipose tissue, skin, muscle, and other tissues. Then, how Tregs are generated, home, and are attracted to and remain resident in the tissue are discussed. Finally, how an increased understanding of these tissue Tregs might guide clinical treatment is discussed.

## Introduction

The immune system includes immune organs and their cells and molecules; it has two main immune responses: innate and adaptive immunity. Regulatory T cells (Tregs), usually referred to as Foxp3^+^CD4^+^ Tregs, are a unique type of immunosuppressive cell in the immune system. Tregs participate in regulating most immune responses and are significant in many physiological processes and diseases, such as immune tolerance, autoimmune diseases, and tumors. It is now clear that Tregs have a strong immune effect in immune tissues, and an effect in maintaining the homeostasis of non-immune tissues (Mueller and Mackay, 2015). The presence of Tregs in visceral adipose tissue (VAT) (Feuerer et al., 2009), muscle (Burzyn et al., 2013), and skin, and the expression and transcription in these cells are different from those in conventional Tregs (Burzyn et al., 2013). The establishment of a tissue-resident immune system makes it possible to respond more quickly to disturbances in local homeostasis during significant diseases, such as infection and foreign matter (Mueller and Mackay, 2015). Therefore, understanding the features of Tregs in tissues is crucial for determining the regulatory mechanism of maintaining homeostasis and using this type of Treg for treatment (Table 1).

**TABLE 1:** Location and functions of tissue Tregs.

Tissue	Location	Functions	References
Visceral adipose tissue	In crown-like structures embracing macrophages	Tregs proliferation and differentiation	Kolodin et al., 2015; Molofsky et al., 2015
		Lipid metabolism and glucose uptake	Feuerer et al., 2009; Kolodin et al., 2015; DiSpirito et al., 2018
		Insulin resistance and sensitivity	Cipolletta et al., 2012; Molofsky et al., 2015; Zhao et al., 2020
		Tissue repair	Kolodin et al., 2015; Molofsky et al., 2015; Delacher et al., 2021
		Anti-inflammation	Feuerer et al., 2009
Skin	Near the hair follicles	HF circulation and hair regeneration	Ali et al., 2017
		Immune tolerance	Yamazaki et al., 2014
		Skin maintenance and reparation	Malhotra et al., 2018; Harrison et al., 2019; Shime et al., 2020
		Stem cell differentiation	Nosbaum et al., 2016
		Antifibrosis	Kalekar et al., 2019
Skeletal muscle	In the connective tissue sheath	Tregs proliferation and maintenance	Kuswanto et al., 2016
		Muscle reparation	Burzyn et al., 2013
		Muscle regeneration	Villalta et al., 2014; Kuswanto et al., 2016
		Downregulate Tregs function	Schenk et al., 2011; Gazzerro et al., 2015
Brain	At the site of injury	Foxp3 regulation and proliferation	Gadani et al., 2015; Garg et al., 2019; Ito et al., 2019; Shi et al., 2021
		Tregs metabolism	Weinberg et al., 2019; Yue et al., 2019
		Tissue reparation and nerve recoveries	Guo and Luo, 2020
		Differentiation of oligodendrocyte precursor cells	Dombrowski et al., 2017
Placenta	In the decidua	Decrease in Tregs accumulation	Paolino et al., 2021
		Suppress Th1 and Th17 cells	Li et al., 2017; Zhang et al., 2018
		Inflammation	Li et al., 2017; Zhang et al., 2018

## The Phenotypes, Factors, and Function of Tissue Tregs

### VAT Tregs

Adipose tissue is mainly divided into subcutaneous adipose tissue and VAT (Rosen and Spiegelman, 2014). Adipose tissue in the viscera stores excess energy and affects the body’s metabolism (Osborn and Olefsky, 2012). In addition to macrophages and neutrophils, Tregs also located in VAT, or more correctly, in the epididymal adipose depot, mediate cellular immune and metabolic networks, maintain the unique microenvironment of adipose tissue, and show different characteristics from those in lymphoid tissue (Rosen and Spiegelman, 2014).

Visceral adipose tissue Tregs are highly abundant in CD4^+^ T cell compartments in lean mice; Tregs are located among adipocytes, usually in crown-like structures embracing macrophages (Feuerer et al., 2009). In C57BL/6 (B6) mice, Tregs in the spleen and lymph glands usually remain at approximately 5–15% of CD4^+^ T cells (Feuerer et al., 2009), while VAT Tregs can reach a peak of 40–80% of CD4^+^ T cells (Cipolletta et al., 2015). However, age-related Foxp3^+^CD4^+^ Treg accumulation does not happen in other white adipose tissues, because obesity-associated metabolic disorders and inflammation are usually not connected to these sites (Tran et al., 2008). Moreover, unlike lymphoid Tregs, which express a wide variety of T cell receptors (TCRs), VAT Tregs express more restricted TCR lineages (Kolodin et al., 2015). With limited TCR diversity in mice, the α amino acid sequence of the complementary determinant region (CDR3) from VAT Tregs is tautologically generated by various nucleotide sequences, indicating antigen selection (Feuerer et al., 2009). VAT Tregs identify one or more unknown peptide antigens that bind to major histocompatibility (MHC) II on the surfaces of VAT antigen-presenting cells, and this process is vital for their accumulation (Kolodin et al., 2015; Li et al., 2018). More importantly, compared to Tregs in immune organs, VAT Tregs obtain a unique transcriptome, which is involved in homeostasis and function (Feuerer et al., 2009; Cipolletta et al., 2015). Diverse genes have different regulatory roles in these Tregs and common Tregs, including those encoding transcription factors [peroxisome proliferator-activated receptor gamma (PPARγ)] (Cipolletta et al., 2012), chemokine and chemokine receptors [C-C motif chemokine receptor 2 (CCR2) and CCR4], cytokines, and cytokine receptors [interleukin (IL)-10 and IL-1 receptor-like 1 (ST2)] (Feuerer et al., 2009; Cipolletta et al., 2015; Han et al., 2015), as well as an interesting group related to lipid metabolism [low-density lipoprotein receptor(LDLR) and diacylglycerol *O*-acyltransferase (DGAT)] (Cipolletta et al., 2012; DiSpirito et al., 2018). And their increase might reflect additional local adaptation to the lipophilic, hypoxic adipose-tissue environment. Although there is overlap in gene expression between activated and VAT Tregs (cluster of differentiation 44 and nuclear receptor subfamily 4 group A member 1) to a certain degree, a large part of these characteristic genes is not simple activation-related genes (Li et al., 2018). Similar to Foxp3, PPARγ is also required to drive the specific phenotype of VAT Tregs and their accumulation, the activation of TCRs is a necessary and sufficient condition to induce PPARγ expression (Cipolletta et al., 2012; Li et al., 2018). Recent research shows that a PPARγ^lo^ Treg population in the spleen contains precursors of VAT Tregs and produces not only VAT Tregs but also other tissue Tregs, such as liver and skin (Li et al., 2021). The last feature of VAT Tregs is growth factor dependence, especially related to IL-33 and IL-33 receptor ST2 (Han et al., 2015; Kolodin et al., 2015). The homeostasis of VAT Tregs is highly dependent on the IL-33-ST2 axis; total blockade of IL-33 or ST2 results in a severe decrease in these Tregs, while Tregs in immune organs are not affected (Kolodin et al., 2015; Molofsky et al., 2015). In addition, IL-33, which induces the differentiation of Tregs *in vitro*, promotes Tregs accumulation by increasing the expansion and activation of Group 2 innate lymphoid cells (ILC2s) and the interaction of T cell costimulatory factor (ICOS) and ICOS ligand (Molofsky et al., 2015). VAT mesenchymal stromal cells (MSCs) balance immunocyte numbers by secreting IL-33, but they do not encode ST2. VAT Tregs and ILC2 express the highest levels of ST2, and, therefore, VAT Tregs are upregulated *via* MSCs and maintain IL-33-expressing stromal cells in a negative feedback loop (Spallanzani, 2021). Tregs have been reported to participate in tissue repair in many researches. These Tregs often express killer cell lectin-like receptor subfamily G1 (klrg1) and ST2 and are found in almost all non-lymphoid tissues. The transcription factor BATF can drive its phenotypic differentiation, which may be the central factor in inducing Tregs tissue repair (Delacher et al., 2020, 2021).

Visceral adipose tissue Tregs regulate the homeostasis of adipose tissue and metabolism; for mice before age 30–40 weeks, VAT Tregs have obvious insulin-sensitizing effects (Feuerer et al., 2009; Ilan et al., 2010; Eller et al., 2011). The transcription factor Zbtb7b suppresses the expression of the soluble form of ST2 (sST2) by inhibiting NF-κB signaling. Mechanistically, sST2 weakens IL-33 signaling and destroys Treg/ILC2 homeostasis in adipose tissue, thus aggravating obesity-associated insulin resistance in mice (Zhao et al., 2020). The PPARγ agonist pioglitazone (Pio) is a type of insulin sensitizers that prevents the loss of VAT Tregs in the course of obesity. The insulin-sensitizing effect of Pio is more than partly due to its function on VAT Tregs, as it is much less valid in mice fed a high-fat diet that lacks PPARγ (Cipolletta et al., 2012). Despite its accumulation, PPAR-γ is also involved in expressing other essential genes involved in Treg cell differentiation, including *Il2*, *Il10*, signal transducer and activator of transcription 5A, *Il33*, and *Il1rl1*, which are not affected by high-fat feeding (Van Herck et al., 2020). The regulation of VAT Tregs through IL-33 also supports the insulin sensitization of Tregs (Vasanthakumar et al., 2015). Although most studies have demonstrated that VAT Tregs enhance insulin sensitivity in lean mice less than 30–40 weeks, VAT Tregs might accelerate insulin resistance in lean mice more than 50 weeks old (Bapat et al., 2015). In this aging model, the mechanism by which VAT Tregs promote insulin resistance is unclear, although the transforming growth factor (TGF)-β pathway is involved (Bapat et al., 2015). The combination of the agonist Fat1562 (one type of surrogate peptide that stimulates clones of VAT Tregs) and anti-tumor necrosis factor (TNF)-α significantly increases the number of VAT Tregs and improves insulin sensitivity in severely obese mice (Fernandes et al., 2020). Androgen also promotes the expansion of the stromal cell population, which produces IL-33, and then recruits Tregs through an inflammatory response (Habrylo and Rosenblum, 2020) depending on the B lymphocyte-induced maturation protein-1 transcription factor (Vasanthakumar et al., 2020). Compared with lymph node Tregs, VAT Tregs release more IL-10, decrease insulin resistance and express TNF-Rs, which may explain why VAT Tregs are more fragile (Feuerer et al., 2009). Moreover, VAT Tregs maintain the balance between anti-inflammatory and pro-inflammatory macrophages, promoting the differentiation of the former and inhibiting the latter (Lynch et al., 2015; Li et al., 2018). TNF-α has been shown to inhibit Glut4-mediated glucose uptake in adipocytes, and this effect can be reversed by IL-10. Since VAT Tregs release abundant IL-10 in adipose tissue, they can suppress inflammatory genes expression, block the downregulation of insulin-dependent cytokines, and reverse the down-regulation of the glucose transporter GLUT4 *via* TNF-α (Feuerer et al., 2009).

### Skin Tregs

As the main barrier organ in close contact with the outside world, the skin is composed of the epidermis and dermis. There are some crosstalks between epithelial cells and immune cells, which can balance between anti-inflammatory and pro-inflammatory immune responses. In adult mouse skin, 20–60% of CD4^+^ T cells are Tregs, and in adult human skin, they comprise about 20% (Scharschmidt et al., 2015). Most mouse Tregs are distributed near the hair follicles (HFs), as are human Tregs (Sanchez Rodriguez et al., 2014; Ali and Rosenblum, 2017). Research shows that mice with dysfunctional Tregs will die of fulminant systemic inflammation at a young age, with obvious dermatitis and hair loss, reflecting the importance of Tregs for inhibiting inflammation (Yang et al., 2015).

The skin Treg bank mainly consists Tregs expressing GATA3, neuropilin 1 (Nrp1), and Helios, which account for 80% of the total Tregs (Wohlfert et al., 2011; Delacher et al., 2017; Malhotra et al., 2018). Skin Tregs have several subsets: Nrp1^*high*^, CD39^+^CD25^high^, CD39^+^Granzyme B, or CD39^+^CTLA-4. Tregs resident in the skin displayed high expression of CD25, CD39, and CCR5 (Ikebuchi et al., 2019). Compared with other immune cells, the Notch signaling ligand Jagged1 (Jag1) and mitochondrial protein arginase 2 (Arg2) are mainly expressed in skin Tregs (Ali et al., 2017; Lowe et al., 2019). Mice lacking GATA3 or RORα in Tregs develop type 2 skin inflammation spontaneously or after challenge (Malhotra et al., 2018; Harrison et al., 2019). In a study of patients with psoriasis, it is found that IL-21 up-regulates RORγt expression and down-regulates the expression of Foxp3, increases secretion of IL-17A and IL-22, and finally induces T-helper 17 (Th17) and Tregs imbalance and promotes inflammation in psoriasis (Shi et al., 2019). Tregs can also promote the repair of skin trauma by the expression of epidermal growth factor receptor (EGFR) (Nosbaum et al., 2016) and are involved in stem cell differentiation in the skin. The lack of EGFR can lead to delayed wound closure and increase accumulation of pro-inflammatory macrophages (Nosbaum et al., 2016). In addition, the tolerance to resident microbiota in the skin depends on Tregs and is connected to the rapid influx of Tregs into the skin during neonatal development (Scharschmidt et al., 2015). In aseptic mice, neonatal skin Tregs decrease by 20% (Scharschmidt et al., 2017). The microbiota promotes the production of HFs derived CCL20, which is the ligand for CCR6 (the skin-homing receptor), promoting the migration of skin Tregs to neonatal skin. Additionally, the maintenance of CD4^+^ and CD8^+^ immune cells in mouse skin has been proven to be dependent on IL-7 derived from keratinocytes (Adachi et al., 2015). FuT7, as an enzyme that promotes the binding of E-selectin and P-selectin, is necessary for the optimal transport of Tregs to the skin, and their retention also seems to require IL-7 rather than IL-2 (Gratz et al., 2013). Thrombospondin-1 (TSP-1) served as the barrier preventing blood vessels into the dermis, is now considered an essential factor in inhibiting Th17 and Treg cell differentiation through the interaction with CD47. In mice, using a CD47-binding TSP-1 peptide increases Foxp3 expression and relieves clinical symptoms of Sjögren syndrome-associated dry eye (Rodriguez-Jimenez et al., 2019). In addition to specific cytokines, Treg cells also interact with other skin resident cells to maintain tissue homeostasis. Fibroblasts can preferentially induce CFSE-labeled purified CD25^hi^CD4^+^ T cells to proliferate in the skin in a contact-dependent manner (Clark and Kupper, 2007); skin Tregs also inhibit the activation of myofibroblasts, which might suppress excessive scar formation during wound healing (Boothby et al., 2020). Moreover, acute and chronic Treg depletion leads to fibrogenic myofibroblasts accumulation, the upregulation of fibrogenic genes, and the down-regulation of IL-10 production and anti-fibrogenic genes (Kalekar et al., 2019).

As mentioned, skin-resident Tregs are mainly distributed around HFs. HFs cycle between growth arrest (telogen) and activation (anagen), processes that are mediated by hair follicle stem cells (HFSCs) in the bulge region of the HF and Tregs have recently been shown to have effect on HF circulation and hair regeneration (Muller-Rover et al., 2001). In clinical trials, 80% of treated patients was observed successful hair regeneration, which may be linked to increased Tregs accumulation in the lesional scalp skin (Castela et al., 2014). Immunophenotypic analysis showed that the number and activation of Tregs in the skin were closely related to specific stages of the HF cycle (Ali et al., 2017). Tregs show a highly activated phenotype in telogen skin, whereas the spectrum-specific depletion of Tregs leads to a significant decrease in HF regeneration. In terms of mechanism, it has been found that the expression of Jag1 in Tregs promotes the HF cycle by improving the activation and differentiation of HFSCs (Ali and Rosenblum, 2017). Moreover, skin Tregs promote HFSC differentiation by controlling the local inflammatory environment, especially preventing CXCL5 mediated Th17 over secretion and neutrophil responses (Mathur et al., 2019). Ultraviolet B irradiation is a stimulus for the skin and results in many of Nrp1^+^ Tregs, which is the main reason for skin immune tolerance (Yamazaki et al., 2014). Skin dendritic cells (DCs) have the unique ability to transform the inactive form of sunlight-derived vitamin D into its bioactive metabolite 1,25-(OH)2-D3. Vitamin D3-induced CD141 dermal DCs resident in human skin preferentially expands Tregs that inhibit skin inflammation *in vivo*. Ultraviolet B light can also expand skin Tregs expressing proenkephalin (PENK), an endogenous opioid precursor, and amphiregulin (Areg), thus supporting wound healing (Shime et al., 2020).

### Skeletal Muscle Tregs

The basic components of skeletal muscle are myofibres, muscle progenitor cells, commonly known as satellite cells, and fibro/adipogenic progenitors (FAPs). Nerves and blood vessels are among the fibers, and they are bound together and surrounded by connective tissue. Leukocytes comprise a small component based on the histological observation of healthy skeletal muscle and have been neglected for a long time (Martinez et al., 2010; Villalta et al., 2014; Wang et al., 2015). Although there are various cell types, including CD8^+^ cytotoxic T cells, Tregs, neutrophils, and eosinophils, each population accounts for only a small part of the total number of leukocytes in healthy muscle. Most of the white blood cells in the muscle are located in the connective tissue sheath around the whole muscle or near blood vessels (Brigitte et al., 2010). Similar to satellite cells, resident macrophages are inactive in stable conditions, but active when muscles are used or damaged, which is useful for regeneration (Krippendorf and Riley, 1993). Traditional Tregs also have great effects on muscle, the loss of which influences muscle repair and regeneration (Krippendorf and Riley, 1993).

The difference between skeletal muscle Tregs and lymphoid Tregs is mainly related to their representation, TCR spectrum, and transcriptome (Burzyn et al., 2013). Foxp3^+^CD4^+^ Tregs exist in muscle and expand quickly after mild freezing injury or severe injury caused by the injection of cardiac toxin, reaching 60% of CD4^+^ T cell subsets. In MDX or dysferlin-knockout mouse models, Foxp3^+^CD4^+^ cell populations are also abundant in muscles, but not in lymphoid organs (Burzyn et al., 2013; Villalta et al., 2014). The proliferation of skeletal muscle DCs is a reaction to acute and chronic injury, and some reports speculate that these skeletal muscle Tregs may respond to local antigens (Burzyn et al., 2013; Kolodin et al., 2015). The clonal duplication of conventional T cells in damaged muscle is also observed, but there is a delay compared to Treg amplification. The expression of Foxp3^+^CD4^+^ Tregs in human and mouse immune organs increases with time (Lages et al., 2008); nonetheless, they are significantly poor in damaged skeletal muscles of old mice (Kuswanto et al., 2016). Data on malnutrition and aging muscle suggest that Tregs attempt to maintain skeletal muscle homeostasis (Burzyn et al., 2013; Villalta et al., 2014). The transcriptome of skeletal muscle Tregs is easily differentiated from that of common Tregs, because the expressions of their genes encoding transcription factors, chemokines and chemokine receptors are different, such as *Ccr2*, *Il10*, and *Il1rl1* (Burzyn et al., 2013; Cipolletta et al., 2015).

The IL-33-ST2 axis is the crucial regulator of Tregs in skeletal muscle and VAT; the main cells producing high levels of IL-33 are one kind of FAPs in muscles (Kuswanto et al., 2016), which help accumulate Tregs during injury by promoting proliferation and reducing lymphatic outflow, but they do not induce T cell chemotaxis (Kuswanto et al., 2016). The *Il33* transcription level peaks within a few hours after muscle injury, and an increase in the number of IL-33^+^ FAPs occurs later. Similarly, in aged mice, the levels of *Il33* transcripts and IL-33^+^ FAPs are decreased; however, when replenishing IL-33, the number of Tregs increases and muscle regeneration is enhanced in aged mice (Kuswanto et al., 2016). Mouse skeletal muscle mesenchymal stromal cells (MmSCs) also link nerves, IL-33, and Tregs; MmSCs that produce IL-33 are not only structurally adjacent to the fiber nerve tracts and sensory neurons, but also encode nerve-related genes and affect Treg accumulation through calcitonin-gene-related peptide (Wang et al., 2020). IL-33 acts on Tregs containing the ST2 receptor encoded by the *Il1rl1* gene; compared to that of Tregs in lymphoid tissue, *Il1rl1* is one of the genes that is upregulated in Tregs isolated from damaged muscle. In ST2-deficient Tregs, *Il1rl1* accumulation is impaired, the clearance efficiency of muscle infiltration is reduced, and muscle regeneration is delayed (Kuswanto et al., 2016). Muscle Tregs express high levels of Areg and support muscle regeneration by acting directly on satellite cells. Areg treatment normalizes the evolution of the muscle transcriptome during muscle repair and promotes myogenic differentiation *in vitro* (Burzyn et al., 2013). Treg loss during muscle regeneration slows down repair, prolonging inflammation, and interfering with the expression of myogenic transcription factors, similar to that with the loss of F4/80^+^ macrophages (Burzyn et al., 2013). These changes could be partly due to the interruption of macrophage phenotype regulation, which weakens the normal transformation of the macrophage phenotype from M1 to M2. The possible function of IL-10 (Villalta et al., 2014) is mainly due to its high level of expression in Tregs and its currently known effect on myeloid cells in regenerated muscle (Burzyn et al., 2013). Tregs are detected in the skeletal muscle of dystrophin-deficient mice, which is a model of human Duchenne muscular dystrophy (Burzyn et al., 2013; Villalta et al., 2014). In addition, ATP released by necrotic muscle fibers and inflammatory cells inhibits Tregs by activating purinergic P2X receptors; blocking the extracellular ATP-P2X purinergic signaling pathway results in an increase in the functions of Tregs in an inflammatory response and the progression of a malnutrition phenotype (Schenk et al., 2011; Gazzerro et al., 2015).

### Brain Tregs

Regulatory T cells play an important role in regulating immune response in the central nervous system (CNS). Tregs deficiency is associated with increased disease progression in Alzheimer’s disease, traumatic brain injury, and stroke (Machhi et al., 2020). In the study of experimental autoimmune encephalomyelitis (EAE), it was found that the depletion of Tregs aggravates the disease and prevents recovery (Koutrolos et al., 2014). Unlike other non-lymphoid tissue such as VAT, intestines, or skin, under steady-state conditions, there are virtually no Tregs in the CNS. However, in the event of trauma or inflammation in the CNS (such as hypoxia or stroke), Foxp3^+^ Tregs will stay in the CNS for a long time and may even establish a resident Treg cell population (Korn et al., 2007; Dombrowski et al., 2017). By analyzing the Treg cells infiltrating the brain after acute cerebral ischemic injury, it was found that these Tregs encode high levels of IL-10, Areg, ST2, and PPARγ, so they seemed to be related to VAT and skeletal muscle Tregs at a transcriptional level (Garg et al., 2019; Ito et al., 2019).

Recent studies have shown that TCR/Irf4 signaling and NF-κB signaling are independent signals needed to establish the transcriptional program in effector Tregs (eTregs), and the transcriptional modifier Blimp1 is the main regulator (Cretney et al., 2011; Vasanthakumar et al., 2017; Rosenbaum et al., 2019). At the mechanistic level, Blimp1 indirectly controls Foxp3 by inhibiting the expression of the methyltransferase DNMT3A in Tregs in inflammation (Garg et al., 2019). The ablation of mitochondrial transcription factor A (TFAM) can lead to a change in the metabolic level in Tregs and decrease the activity of the demethylase Tet enzyme. Then, the demethylation state of conserved non-coding regions 2 (*Cns2*) cannot be maintained, ultimately affecting the stability of Tregs (Weinberg et al., 2019; Yue et al., 2019). In contrast, DNA methyltransferases (DNMT1 and DNMT3A) can methylate *Cns2* (or other CpG islands) to inhibit Foxp3 expression. Given the high expression of ST2 in brain Tregs, which is structurally expressed in the CNS, and that Tregs cannot be generally expanded in the damaged ischemic brain of IL-33/ST2-deficient mice, IL-33 might be an important molecule to replace IL-2 in mediating the survival of brain Tregs after inflammation (Gadani et al., 2015). It has been confirmed that IL-33 can increase the number of Tregs in ischemic brain tissue. The increased Tregs produce Areg and activate EGFR in neurons, which is helpful to improve the prognosis (Guo and Luo, 2020). Another possible influencer of Tregs is the neurotransmitter 5-hydroxytryptamine (5-HT). Its receptor 5-HT7 specifically upregulates Tregs gathered in the ischemic brain. Studies have shown that 5-HT or the inhibition of its uptake can increase the number of Tregs in the brain (Ito et al., 2019). In the toxic demyelination model, Tregs have been shown to support the regeneration of myelin *via* promoting the differentiation of oligodendrocyte precursor cells in the brain by CCN3 (a growth regulatory protein) (Dombrowski et al., 2017). Additionally, brain Tregs upregulate certain CNS-specific genes, such as neuropeptide Y (*NPY*), *PENK*, 5-HT7, and arginine vasopressin receptor (*AVPR1A*) (Ito et al., 2019). Particularly, the increased expression of the EGFR ligand modulin in Tregs recruited by the CNS seems vital for processes associated with nerve recoveries, such as the inhibition of astrocyte proliferation, neuron dysfunction, and neurotoxic gene expression (Ito et al., 2019). The latest research reports that Treg-derived osteopontin plays a role through the integrin receptors on microglia, enhances the repair activity of microglia, thus promotes the formation of oligodendrocytes and the repair of white matter. After a stroke, increasing the number of Tregs by delivering an IL-2:IL-2 antibody complex can improve the integrity of white matter and save neurological function for a long time (Shi et al., 2021).

### Other Tissues

**FIGURE 1:**
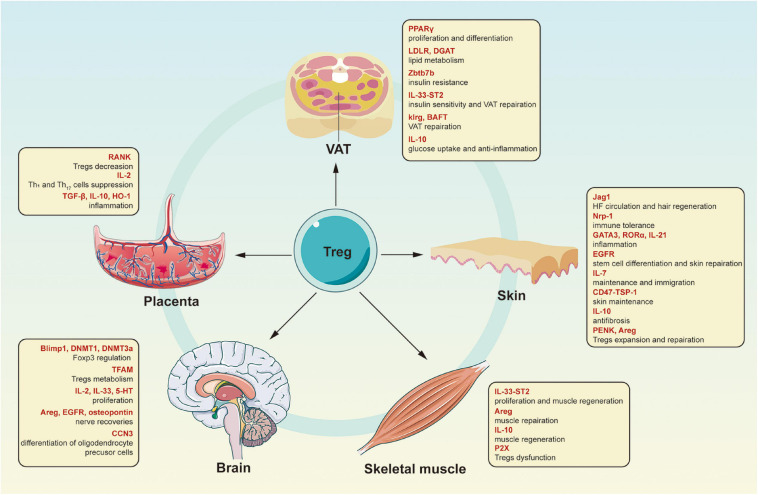
Characteristics of different kinds of tissue Tregs. Tregs are not only critical for affecting immune response, but also for maintaining non-lymphoid tissues homeostasis *via* different cytokines or interacting with other tissue cells. Tregs, regulatory T cells; PPARγ, peroxisome proliferator-activated receptor gamma; LDLR, low-density lipoprotein receptor; DGAT, diacylglycerol *O*-acyltransferase; Zbtb7b, Zinc finger and BTB domain-containing protein 7B; IL-33, interleukin-33; ST2, IL-1 receptor-like 1; klrg1, killer cell lectin-like receptor subfamily G1; BATF, basic leucine zipper ATF-like transcription factor; IL-10, interleukin-10; Jag1, the Notch signaling ligand Jagged1; Nrp1, neuropilin 1; GATA3, GATA binding protein 3; RORα, retinoic acid-related orphan receptor alpha; IL-21, interleukin-21; EGFR, epidermal growth factor receptor; IL-7, interleukin-7; TSP-1, thrombospondin-1; PENK, proenkephalin; Areg, amphiregulin; P2X, purinergic receptor P2X; Blimp1, B lymphocyte-induced maturation protein 1; DNMT1, DNA methyltransferases 1; DNMT3a, DNA methyltransferases 3 alpha; TFAM, mitochondrial transcription factor A; IL-2, interleukin-2; 5-HT, 5-hydroxytryptamine; CCN3, cellular communication network factor 3; RANK, NF-κB ligand; TGF-β, transforming growth factor beta; HO-1, heme oxygenase 1.

Tissue Tregs located in the VAT, skin, and skeletal muscle are the most characteristic Treg communities; however, other tissues also contain Tregs (Figure 1). A special Treg population exists in the placenta; Tregs in the decidua of mouse placenta express alloantigens from their father, dependent on *Cns1* (Samstein et al., 2012). When pregnant with offspring of allogeneic (not syngeneic) males, female mice with *Cns1* extension lacking Foxp3 show increased fetal absorption and placental immune cell infiltration (Rowe et al., 2012). At the same time, Tregs in the human placenta seem to be essential to control inflammation in early pregnancy and establish an acceptable decidual environment through its anti-inflammatory effect (Erlebacher, 2013). According to former researches, there are now three distinct decidual CD4^+^ Treg types in healthy pregnancies with a regulatory phenotype and the ability to suppress T cell responses: CD25^hi^Foxp3^+^, PD1^hi^IL-10^+^, and TIGIT^+^Foxp3^dim^. The loss of NF-κB ligand (RANK) in the thymus epithelium of mice leads to a decrease in Treg accumulation in the placenta, increasing the possibility of abortion (Paolino et al., 2021). During an absence of Tregs, several crucial biological processes related to embryonic development and cell metabolism are out of balance, which may cause fetal growth disorders and premature delivery in the third trimester of pregnancy (Gomez-Lopez et al., 2020). Tregs suppress the activation and function of T-helper 1 (Th1) and Th17 cells through sequestering IL-2 and other inhibitory mechanisms, and control inflammation by releasing TGF-β, IL-10, and heme oxygenase 1 (HO-1) by interacting with DCs and uterine natural killer cells (Li et al., 2017; Zhang et al., 2018). For example, the activation of invariant natural killer T (iNKT) cells decreased the frequency of decidual Tregs, the production of IL-10 and TGF-β, and suppressive Tregs activity, indicating that iNKT cells may have a role in inflammatory pregnancy loss *via* suppression of decidual Tregs function. The ability of Tregs to transform into T effectors in infection, severe inflammation, or interruption of fetal development confers the ability to terminate the pregnancy and ensures maternal survival (D’Addio et al., 2011).

## Occurrence and Development of Tissue Tregs

**FIGURE 2:**
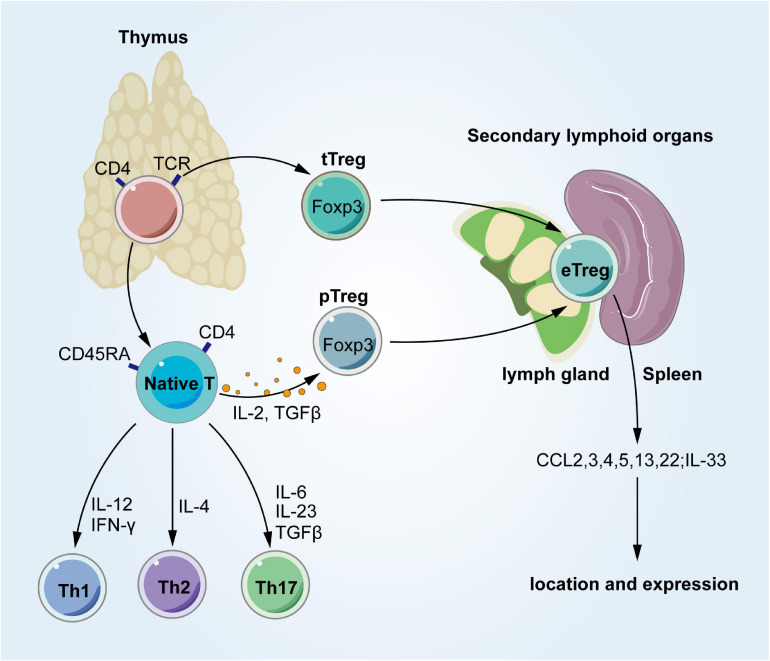
Occurrence and development of tissue Tregs. Derived from the normal thymus, Tregs have two different ways to develop. In the thymus, CD4^+^ thymocytes turn into tTregs by TCRs. In periphery, Tregs occur with IL-2 and TGF-β. These Tregs stay in SLO, and then migrate into tissues once being stimulated. Tregs, regulatory T cells; tTregs, thymic Tregs; pTregs, peripheral Tregs; TCR, T cell receptors; Th1, T-helper 1; Th2, T-helper 2; Th17, T-helper 17; IFN-γ, interferon-gamma; TGF-β, transforming growth factor beta; IL-12, interleukin-12; IL-4, interleukin-4; IL-6, interleukin-6; IL-2, interleukin-2; IL-23, interleukin-23; CCL, chemokine (C-C motif) ligand.

Regulatory T cells develop in the thymus, where there are two different pathways (Josefowicz et al., 2012). Thymic Tregs (tTregs) differentiate into Foxp3^+^ Tregs in the thymus after TCR-recognized autoantigen binding. In contrast, peripheral Tregs (pTregs) stay away from the thymus as naive CD4^+^ T cells and differentiate into Foxp3^+^ Tregs in secondary lymphoid organs (SLOs) after recognizing their homologous antigens (Figure 2). Although it is not a strict criterion for differentiation, Helios and the membrane protein Nrp1 expressed in tTregs (not pTregs) may be used to distinguish these Treg subsets (Nutsch et al., 2016). Tregs with activated phenotypes, such as ICOS, glucocorticoid-induced TNFR family related, and IL-10, exist in SLOs and are called effector Tregs (eTregs) (Cretney et al., 2013). Tregs respond to tissue homing signals and then migrate to tissues after stimulating TCRs in SLOs (Wei et al., 2006). eTregs are linked to phenotypic specialization and enhanced migration to tissues, which might represent the intermediate developmental stage of Tregs from the thymus to tissues. Several tissue features have been identified in SLO Tregs, such as a few VAT Treg signatures in spleen Tregs (Vasanthakumar et al., 2015; Li et al., 2018). SLO Tregs also increase the expression of transcription factors related to effector T cells, such as T-box expressed in T cells (T-bet), GATA3, STAT3, and RoRγt (Wang et al., 2011; Wohlfert et al., 2011; Cretney et al., 2013; Schiering et al., 2014; Ohnmacht et al., 2015; Sefik et al., 2015). Although studies using parabiosis show that Tregs are not easily recirculated, they may be continuously supplemented from circulating precursor cells, such as naive Tregs (Lynch et al., 2015). And regardless of its origin, the tissue tree must reach the appropriate target tissue and survive. A model has been proposed for the development of Tregs that reside in the VAT, colon, and damaged muscle, and has been shown to localize and expand in tissues through a process dependent on IL-33 (Cipolletta et al., 2012; Schiering et al., 2014; Kolodin et al., 2015; Vasanthakumar et al., 2015; Kuswanto et al., 2016). Data show that several components have effects, such as a TCR-MHCII peptide, chemokine-chemokine receptors, and cytokine-cytokine receptor interactions. One of these is G protein-coupled receptor 15, which directs Tregs to the colon lamina propria (Kim et al., 2013). Some of these tissue Treg activities regulate inflammatory cells nearby (Feuerer et al., 2009; Cretney et al., 2013), and they also directly affect the surrounding parenchymal cells (Feuerer et al., 2009; Burzyn et al., 2013; Schiering et al., 2014; Arpaia et al., 2015). Some of the different transcripts in tissue Tregs are dedicated to controlling the underlying pathological process of their release in non-immunologic host tissue cells (Luck et al., 2015).

## Concluding Remarks

Regulatory T cells are essential for maintaining tissue homeostasis. Although existing studies have shown Treg mechanisms in related tissues, the antigenic reactivity and metabolic adaptability of Tregs should be further studied, and whether there are corresponding Tregs in other tissues is also worth exploring. Since tissue Tregs have similar transcription factors with other tissue cells, studying the interaction between them (such as the neuron population) will further promote the understanding of the role of tissue Tregs. Using emerging technologies to further reveal new phenotypes and functions of tissue Tregs provides better guidance and direction for the treatment of the increasing number of chronic tissue diseases and immune deficiency diseases.

## Author Contributions

QS and JG conceived the manuscript. QS wrote the manuscript, drew the figures and tables, and was a major contributor in writing. JG and JZ contributed to the organization, suggestions on the content, and editing of the manuscript. QW, XL, ZD, and LL revised the manuscript. All authors read and approved the final manuscript.

## Conflict of Interest

The authors declare that the research was conducted in the absence of any commercial or financial relationships that could be construed as a potential conflict of interest.

## Publisher’s Note

All claims expressed in this article are solely those of the authors and do not necessarily represent those of their affiliated organizations, or those of the publisher, the editors and the reviewers. Any product that may be evaluated in this article, or claim that may be made by its manufacturer, is not guaranteed or endorsed by the publisher.
